# Association of rs3736228 polymorphism in LRP5 gene with bone fracture and osteoporosis: a systematic review and updated meta-analysis

**DOI:** 10.1186/s12891-026-10270-9

**Published:** 2026-07-27

**Authors:** Chi Zhang, Wuhua Liu, Youhua Wang, Yuyi Peng

**Affiliations:** 1 Department of Orthopaedics, Affiliated Jinhua Hospital, Zhejiang University School of Medicine (Jinhua Municipal Central Hospital), Jinhua, China; 2 The Second Retirement Home for Cadres of Shenyang Military Sub-district of Liaoning Province, Shenyang, China; 3 School of Business, Hunan University of Humanities, Science and Technology, Loudi, China; 4 National Center for Chronic and Noncommunicable Disease Control and Prevention, Chinese Center for Disease Control and Prevention, Beijing, China

**Keywords:** *LRP5*, rs3736228, Polymorphism, Fracture, Meta-analysis, Osteoporosis

## Abstract

**Background:**

The low-density lipoprotein receptor-related protein 5 (*LRP5*) gene is a key regulator of bone mineral density and skeletal metabolism. The rs3736228 (Ala1330Val) polymorphism in *LRP5* has been firmly established as a genome-wide significant locus for bone mineral density and fracture risk in large-scale GWAS. However, demographic and clinical subgroups that are frequently underrepresented in GWAS cohorts, such as sex, menopausal status and ethnicity, may have different impact sizes and ideal genetic models. Previous candidate-gene meta-analyses, while informative, did not systematically evaluate these subgroup-specific effects, owing to limited sample sizes and incomplete stratification. Therefore, we conducted this updated systematic review and meta-analysis to provide granular, subgroup-stratified risk estimates that complement existing GWAS evidence.

**Methods:**

This systematic review was conducted in accordance with PRISMA guidelines. A comprehensive electronic search was performed across five databases: PubMed, Embase, Cochrane Library, Web of Science, and Ovid. The literature search was conducted using a combination of Medical Subject Headings(MeSH)terms, such as"osteoporosis, “fracture, “*LRP5*,“and"polymorphism,“along with their related synonyms. We also manually screened reference lists of retrieved articles to identify additional relevant studies. To address heterogeneity, we employed both the Cochran’s Q test and the I²statistic. A fixed-effects model was used when I²<50%and *P* ≥ 0.05; otherwise, a random-effects model was applied. All analyses were performed using STATA software version 17.0, and the odds ratios(ORs)and their corresponding 95%confidence interval(CI)values were calculated using fixed effects or random effects model. We carried out a subgroup analysis to explore the source of heterogeneity according to gender, race, disease, genotyping methods, control resources, female menopause, and HWE balance.

**Results:**

The current meta-analysis involved 14 studies, comprising 16 independent comparisons, including 1,549 patients and 3,345 control subjects. The analyses revealed a significant association between *LRP5* rs3736228 polymorphism and an increased risk of osteoporosis and fractures. Specifically,in the overall analysis, the polymorphism showed a higher likelihood of osteoporosis and fractures in the allelic model [OR=1.17, 95%CI(1.04-1.31), *P*=0.011], homozygous model [OR=1.47, 95%CI(1.07-2.01), *P*=0.018], dominant model [OR=1.44, 95%CI(1.05-2.97), *P*=0.023], and recessive model [OR=1.16, 95%CI(1.01-1.33), *P*=0.017]but not in the heterozygous model [OR=1.34, 95%CI(0.96-1.88), *P*=0.09]. Subgroup analyses showed that the polymorphism was associated with increased risk in males under the allelic(OR = 1.59, 95% CI 1.06–2.39, *P* = 0.026)and dominant models(OR = 1.74, 95% CI 1.11–2.73, *P* = 0.016). In females, risk was elevated in the homozygous(OR = 1.50, 95% CI 1.05–2.14, *P* = 0.027)and recessive models(OR = 1.48, 95% CI 1.04–2.09, *P *= 0.030). In population-based controls, significant associations were observed in the allelic(OR = 1.21, 95% CI 1.06–1.39, *P* = 0.005), homozygous(OR = 1.49, 95% CI 1.06–2.10, *P* = 0.021), dominant(OR = 1.22, 95% CI 1.03–1.43, *P* = 0.017), and recessive models(OR = 1.46, 95% CI 1.04–2.04, *P* = 0.027). No significant associations were found in the hospital-based subgroup. Heterogeneity was partly explained by disease type, publication year, and control source(*P* < 0.05 in multivariate meta-regression).

**Conclusion:**

This updated meta-analysis confirms that the LRP5 rs3736228 polymorphism is significantly associated with osteoporosis and fracture risk, consistent with GWAS evidence, while further providing risk estimates stratified by sex and menopausal status, which are not readily obtainable from GWAS-level summary statistics alone.Notably, the observed associations appeared less robust in males, and premenopausal females, although these subgroup findings should be interpreted with caution owing to limited sample sizes. Nevertheless, further large-scale, prospective studies are warranted to validate these subgroup-specific estimates and to evaluate their potential utility in clinical risk stratification.

**Supplementary Information:**

The online version contains supplementary material available at https://doi.org/10.1186/s12891-026-10270-9.

## Introduction

Osteoporosis, a condition characterized by progressive bone tissue degeneration and low bone density, is becoming increasingly prevalent in the aging population. This condition leads to increased bone fragility and susceptibility to fractures in various bones with severe consequences, such as increased mortality, physical impairment, higher healthcare costs, and poor quality of life [1]. It is estimated that 20%of men and 50% of women, over the age of 50 years, experience a fracture associated with osteoporosis. Moreover, due to changing population demographics, it is projected that the number of individuals with osteoporosis will double in the next two decades [2]. As a multifactorial disease, osteoporosis has a complex etiology that often goes undetected in most individuals affected by it [3]. It is caused by several environmental factors, including age, smoking, physical activity, nutrition, and malabsorption [4]. Beyond these environmental and clinical factors, accumulating evidence from genome-wide association studies (GWAS) has established that specific genetic polymorphisms substantially contribute to interindividual variation in BMD and osteoporosis risk [5, 6]. In particular, polymorphisms in the *LRP5* gene, which plays a key role in the Wnt signaling pathway and bone formation, have been widely studied for their association with osteoporosis susceptibility [7, 8].

The Wnt signaling pathway plays a pivotal role in regulating bone homeostasis, cell differentiation, and bone formation [9]. The Wnt/β-catenin signaling pathway is activated by secreted Wnt glycoproteins, which are released by various cell types, including osteoblasts, endothelial cells, and mesenchymal stem cells. The key components of this pathway include Wnt ligands, the Frizzled receptors, and the *LRP5* co-receptors, which together activate downstream intracellular signaling [10, 11]. *LRP5* acts as a gatekeeper for Wnt responses, enabling the Wnt pathway’s responsiveness. In humans, the *LRP5* gene is located on chromosome 11q13.4, spanning approximately 160 kb, including 22 introns and 23 exons [12]. *LRP5* plays a crucial role in bone growth and health, as well as in blood glucose and lipid metabolism, preventing the reduction in bone production. Moreover, *LRP5* has been associated with early-onset osteoporosis [13–15]. The Ala1330Val(rs3736228, exon 18)polymorphism has been the focus of research and has been demonstrated to impact bone mineral density [7, 16, 17].

At the GWAS level, the association of LRP5 rs3736228 with BMD and fracture risk has been firmly established.In a landmark genome-wide association study, Richards et al. [18] identified this non-synonymous variant as genome-wide significantly associated with lumbar spine BMD (*P* = 6.3 × 10⁻¹²) and femoral neck BMD (*P* = 1.9 × 10⁻⁴), as well as with increased risk of osteoporotic fractures (OR 1.3, 95% CI 1.09–1.52). The GEFOS consortium then carried out the largest meta-analysis to date, combining 17 GWAS and 32,961 participants, and found 56 BMD loci, 14 of which, including LRP5, were also linked to fracture risk [19]. More recently, Trajanoska et al. [20] performed a large-scale fracture GWAS meta-analysis and confirmed LRP5 as one of 15 genetic determinants influencing both BMD and fracture susceptibility.Collectively, these GWAS findings have unequivocally validated LRP5 rs3736228 as a robust genetic determinant of skeletal fragility.

Despite this well-established association, several key questions remain unanswered.First, the effect size and genetic model of this polymorphism may differ across specific demographic and clinical subgroups, such as males, premenopausal women, and different ethnic populations, that have been underrepresented in large GWAS cohorts, which were predominantly composed of European ancestry. Second, the optimal genetic model (allelic, dominant, recessive, or homozygous) for risk estimation may vary by sex and menopausal status, yet no systematic meta-analytic evaluation has comprehensively compared these models across subgroups. Third, new primary studies focusing on these understudied populations have been published in recent years, offering additional data that, when synthesized, could provide more granular and clinically actionable risk estimates than those derived from GWAS-level summary statistics alone.

To address these gaps and complement the existing GWAS evidence, we carried out an updated systematic review and meta-analysis. First, to incorporate the most recent evidence on the *LRP5* rs3736228 polymorphism and osteoporosis risk; Second, to conduct detailed subgroup and sensitivity analyses across demographic and clinical variables; and Third, to generate precise, stratified risk estimates that may inform future genetic association studies and contribute to personalized risk stratification, particularly in populations historically underrepresented in large-scale GWAS.

## Materials and methods

### Literature search and data sources

A comprehensive search was conducted using digital databases, including PubMed, Embase, Cochrane Library, Web of Science, and Ovid. The search utilized a combination of Medical Subject heading(MESH)terms and text words. The key search terms included“osteoporosis”, “fracture”, “*LRP5*”, and“polymorphism”. Moreover, synonyms and other search phrase formats were employed to retrieve all pertinent papers. The literature search was last updated on 11/1/2025. In order to identify additional relevant literature, two independent reviewers individually assessed each title and scrutinized the references and abstracts of relevant studies. This systematic review protocol was prospectively registered on PROSPERO(Registration ID: CRD420261283543).

### Selection criteria

The inclusion criteria included: (1) case-control or cohort or cross-sectional studies previously published in peer-reviewed journals; (2) only studies investigating the association between the *LRP5*(rs3736228)polymorphism SNP with the risk of osteoporosis and fracture considered throughout the meta-analysis; (3) all participants underwent diagnostic examination and fulfilled the clinical criteria for fracture or osteoporosis according to the World Health Organization Osteoporosis Working Group report [21, 22]; (4) articles had to provide raw data and sufficient information to calculate the odd ratio(OR)and its 95%confidence intervals(95%CI); and (5) in case of overlapping data, preference was given to studies with the largest sample size.

The exclusion criteria for this meta-analysis included: (1) certain publications, including letters, editorials, reviews, abstracts, meta-analyses, opinion articles, and conference proceedings, published as published non-original data; (2) unpublished data sources or duplicates; (3) duplicates of publications or studies without extractable numerical data; (4)studies not related to fractures or specific gene polymorphisms; (5) animal experiments; (6) non-case-control designs; and (7) unavailability of raw data even when authors were contacted.

### Quality assessment of included literature

The methodological quality and risk of bias of the included studies were independently evaluated by two reviewers. Initially, each reviewer assessed the studies based on the pre-defined inclusion and exclusion criteria. To ensure consistency and resolve any discrepancies in the quality assessment, reviewer agreement was quantified using the Kappa statistic. Studies were evaluated using the Newcastle-Ottawa Scale(NOS)for case-control studies and the Agency for Healthcare Research and Quality(AHRQ)checklist for cross-sectional studies. The NOS criteria were evaluated based on the study design, content, and interpretation of results or meta-analyses evaluating quality [23]. Three main aspects were assessed: (1)study population selection, 0–4;(2)study population comparability, 0–2;and(3)clinical outcomes,0–3.NOS scores ranged from 0–9;with scores of ≥ 7 considered indicative of good study quality.The AHRQ [24], consisting of 11 items, scored 1 for each item assessed as"yes"and 0 otherwise.The total score was categorized aslow quality(0–3),moderate quality(4–7),and high quality(8–11).Both reviewers independently evaluated the studies based on the previously stated inclusion/exclusion criteria.

### Data extraction

Two researchers(Y.Y.Peng and Chi Zhang)independently performed data extraction using a pre-designed form (see Supplementary Tables 1 and 2). Inter-rater agreement for extracted items was assessed using Cohen’s kappa(κ)statistic. After a rigorous screening of the literature against the inclusion and exclusion criteria, eligible studies proceeded to full data extraction. Any discrepancies between the two extractors were resolved through discussion; if consensus could not be reached, a third senior author was consulted to re-evaluate the data, and the final decision was verified collectively. In cases where reported information was ambiguous or incomplete, the corresponding authors of the original studies were contacted to obtain clarification or the original data. The following data items were systematically extracted from each study: (1)first author’s name, (2)publication year, (3)origin country, (4)ethnicity of the studied population, (5)study design, (6)sex distribution in the studied population, (7)age distribution in the studied population, (8)genotyping technique, (9)type of control group, (10)sample size, (11)genotype distributions, and(12)Hardy-Weinberg equilibrium(HWE)in controls(*r*-value).

### Certainty of evidence

We used the Grading of Recommendations, Assessment, Development and Evaluation(GRADE)approach to rate the quality of evidence for the primary and secondary outcomes.We followed the GRADE handbook to perform the quality assessment.The GRADE pro Guideline Development Tool(GDT)tool was used to generate the results.All assessments were conducted by two investigators independently followed by discussion until an agreement was reached.

### Statistical analyses

The meta-analysis was performed using the following approaches: (i)allelic contrast(T vs.C), (ii)homozygous contrast(TT vs.CC), (iii)heterozygous contrast(*TT vs.*TC), (iv)recessive contrast(TT vs.TC + CC), and(v)dominant contrast(TT + TC vs.CC). To account for multiple comparisons across these five genetic models and reduce the risk of false positives, a Bonferroni correction was applied.Accordingly, the significance threshold for the primary association analyses under these genetic models was adjusted to *P* < 0.01(i.e.,0.05/5).The OR and 95%CI were computed for dichotomous data. The statistical heterogeneity was evaluated using *I*2 andχ2 tests [25, 26], with a significance threshold set at *P* < 0.05. If no heterogeneity was observed(*I*2 < 50%,*P* > 0.05)across the trials, a fixed-effects model was used; otherwise, a random-effects model was employed.

Subgroup analyses were conducted based on race, disease, sex, female menopause, control source, and HWE balance. The sensitivity analyses were performed to evaluate the influence of specific studies on overall estimations. Moreover, Begg’s and Egger’s tests using ocular examination of funnel plots were employed to assess publication bias [27, 28]. The sources of heterogeneity were identified using meta-regression. To evaluate the credibility of statistically significant findings, two additional measures were applied: The Venice criterion [29] were used to grade the credibility of evidence and the false positive reporting probability(FPRP) [30] were used to estimate the probability that a significant finding is a false positive. Statistical analyses were conducted using Stata version 17.0, and all the tests were two-tailed with *P* < 0.05 considered statistically significant, whereas the adjusted threshold of *P* < 0.01 was used to declare statistical significance for the primary genetic model comparisons.

## Results

### Literature search and study attributes

**Fig. 1:**
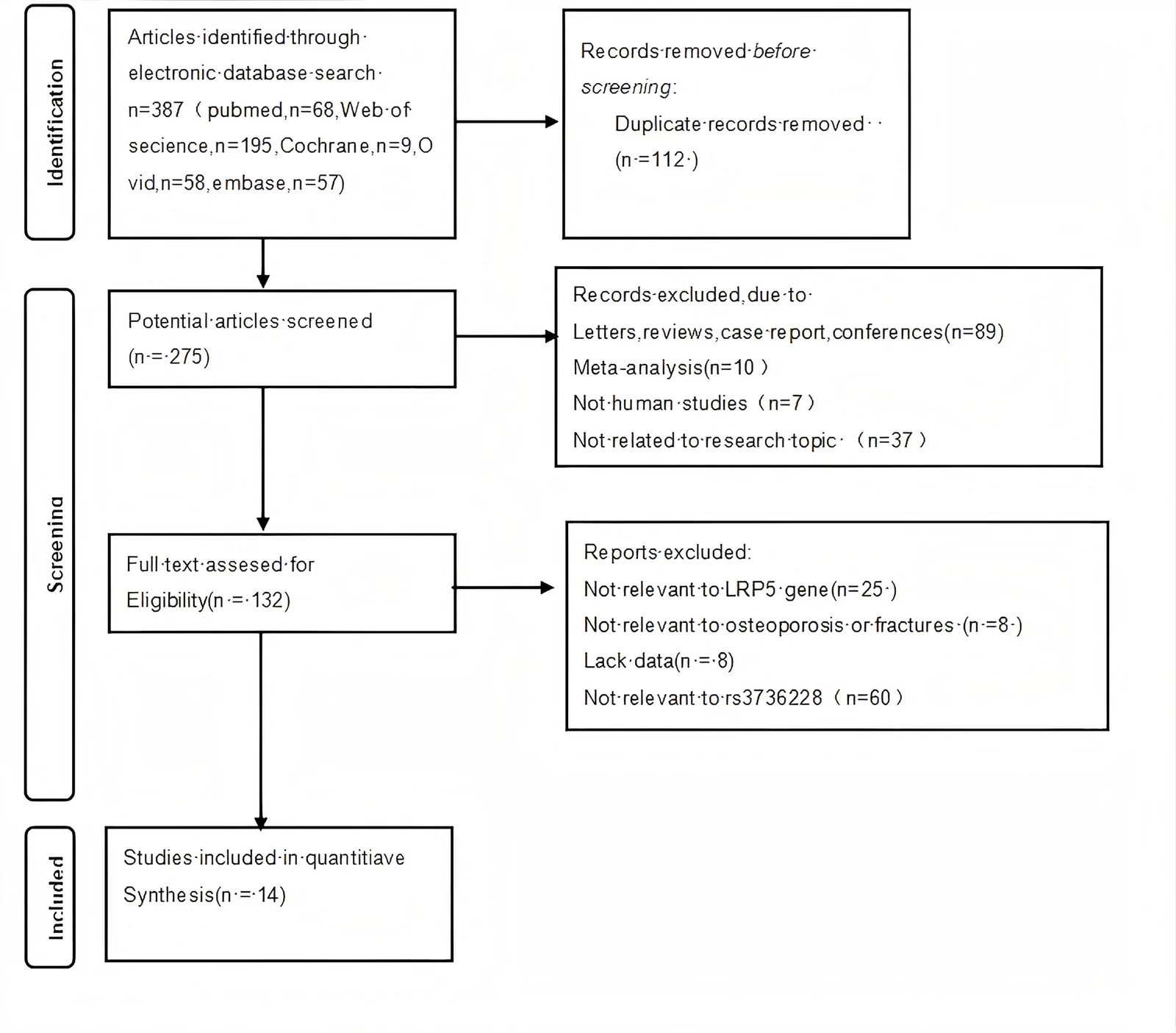
Flow chart shows the selection procedure. Fouteen studies were included in the studies

A total of 387 potentially suitable articles were initially identified through computerized and manual searches.After excluding 112 duplicate studies, the remaining 275 studies were screened for relevance based on title and abstract.Exclusion criteria included, letters, reviews, case reports, conference papers, meta-analyses, and studies not involving human subjects. Subsequently, 132 articles available for full-text review were carefully examined, resulting in the exclusion of 118 papers deemed inappropriate. Ultimately, this meta-analysis included 14 articles having a total of 4894 participants, including 1549 patients and 3345 control subjects. The selection process and primary reasons for exclusion are illustrated in Fig. 1. Each paper included in this study demonstrated a quality level ranging from moderate to excellent quality.

Among the 14 included studies, 4 studies included patients with fractures, while the remaining 10 studies investigated patients with osteoporosis.Additionally, seven studies investigated Caucasians, and another seven studies focused on Asians; three studies included children, while 11 studies targeted adults. Nine studies specifically investigated women, while two studies concentrated on men. Regarding genotyping methods, six studies did not use the TaqMan method(Sequenom, Snapshot, PCR-RFLP, pyrosequencing), while the remaining eight studiesemployed the TaqMan method. Furthermore, three studies did not adhere to the HWE law(*P* < 0.05), while the remaining 13 studies were consistent with the HWE law(*P* > 0.05).Information on the *LRP5* gene SNP(rs3736228 polymorphism)was included to assess the association of *LRP5* gene polymorphic variants with osteoporosis and fracture risk. The genotype frequencies and baseline characteristics of each unique rs3736228 polymorphic SNP are displayed in Tables 1 and 2.

**Table 1:** Baseline characteristics and methodology quality of included studies

Author/year	Race	Area	Diseases	Cases/Controls	Age		Gender(F/M)		Genotyping	NOS	HWE-test
					Cases	Controls	Cases	Contols	Method	Score	
Inass M.2023 [8]	Caucasians	Saudi	OP	12/591	22(20–37)	23(18–40)	12/0	591/0	TaqMan	7	0.7
J Li.2022 [31]	Asians	China	OP	28/26	69.70 ± 7.30	65.60 ± 8.20	28/0	26/0	Sequenom	8	0.42
				55/27	70.50 ± 6.40	66.50 ± 7.50	55/0	27/0			0.28
Masaki.2021 [32]	Asians	Japan	OP	22/125	75.1 ± 10.3	64.0 ± 8.2	22/0	125/0	TaqMan	7	0.28
He bai.2021 [33]	Asians	China	OP	87/100	9.67 ± 1.35	9.34 ± 1.57	60/27	58/42	Snapshot	8	0.05
Alina.2019 [17]	Caucasians	Romania	OP	228/136	65.5 ± 7.39	63.45 ± 8.16	228/0	136/0	TaqMan	8	0.0018
Xuan.2014 [34]	Asians	China	OP	90/78	60.0 ± 3.0	60.5 ± 2.7	90/0	78/0	PCR-RFLP	8	0.04
				90/96	60.0 ± 3.0	60.4 ± 3.2	90/0	96/0			0.25
Falcon.2013 [35]	Caucasians	Mexican	OP	100/217	64.7 ± 10.5	57.4 ± 12.0	100/0	217/0	TaqMan	8	0.95
Liu.2010 [36]	Asians	China	Fracture	284/728	60.1 ± 10.5	54.9 ± 11.2	284/0	728/0	TaqMan	7	0.25
Korvala.2010 [37]	Caucasians	Finland	Fracture	144/240	20.3 ± 1.6	18.9 ± 0.5	0/144	0/240	pyrosequencing	7	0.95
Saarine.2010 [38]	Caucasians	Finland	Fracture	66/235	10.2(4.4–16.8)	**/**	21/45	**/**	pyrosequencing	7	0.96
Furuya.2009 [39]	Asians	Japan	Fracture	119/441	**/**	**/**	119/0	441/0	TaqMan	8	0
Hartikka.2005 [40]	caucasians	Finland	OP	20/88	10.0(4–16)	**/**	9/11		TaqMan	7	0.96
Ferrari.2005 [41]	caucasians	Switherland	OP	78/86	50.7 ± 9.9	49.8 ± 16.1	0/78	0/86	pyrosequencing	8	0.96
Mizuguchi.2004 [42]	Asians	china	OP	126/131	**/**	**/**	126/0	131/0	TaqMan	7	0.88

Two studies (J Li 2022 and Xuan 2014) [31, 34] each contributed two separate comparisons based on different subgroups

*OP *osteoporosis, *F/M *female/male, *NOS *Newcastle-Ottawa Scale, *HWE *Hardy-Weinberg equilibrium

**Table 2:** The distribution of *LRP5* rs3736228 genotypes for postmenopausal osteoporosis and controls

Study	Publication year	Osteoporosis			Controls			Pa
		CC	CT	TT	CC	CT	TT	
Inass M	2023	4	6	2	376	191	24	0.7
J Li	2022	17 34	10 18	1 2	19 20	7 4	0 1	0.42 0.28
Nacano	2021	10	8	4	62	58	5	0.055
He bai	2021	70	15	2	95	4	1	0.0018
Alina	2019	169	56	3	88	47	1	0.04
Xuan	2014	64 57	23 31	3 2	50 60	25 34	3 2	0.95 0.25
Falcon	2013	55	41	4	141	71	5	0.95
Liu	2010	171	99	14	450	245	33	0.96
Saarine	2010	53	6	7	197	18	20	0
Korvala	2010	124	19	1	217	22	1	0.96
Furuya	2009	67	40	12	236	173	32	0.96
Hartikka	2005	13	6	1	50	33	5	0.88
Ferrari	2005	43	33	2	61	23	2	0.92
Mizuguchi	2004	62	53	11	76	48	7	0.87

### Overall analyses

**Fig. 2:**
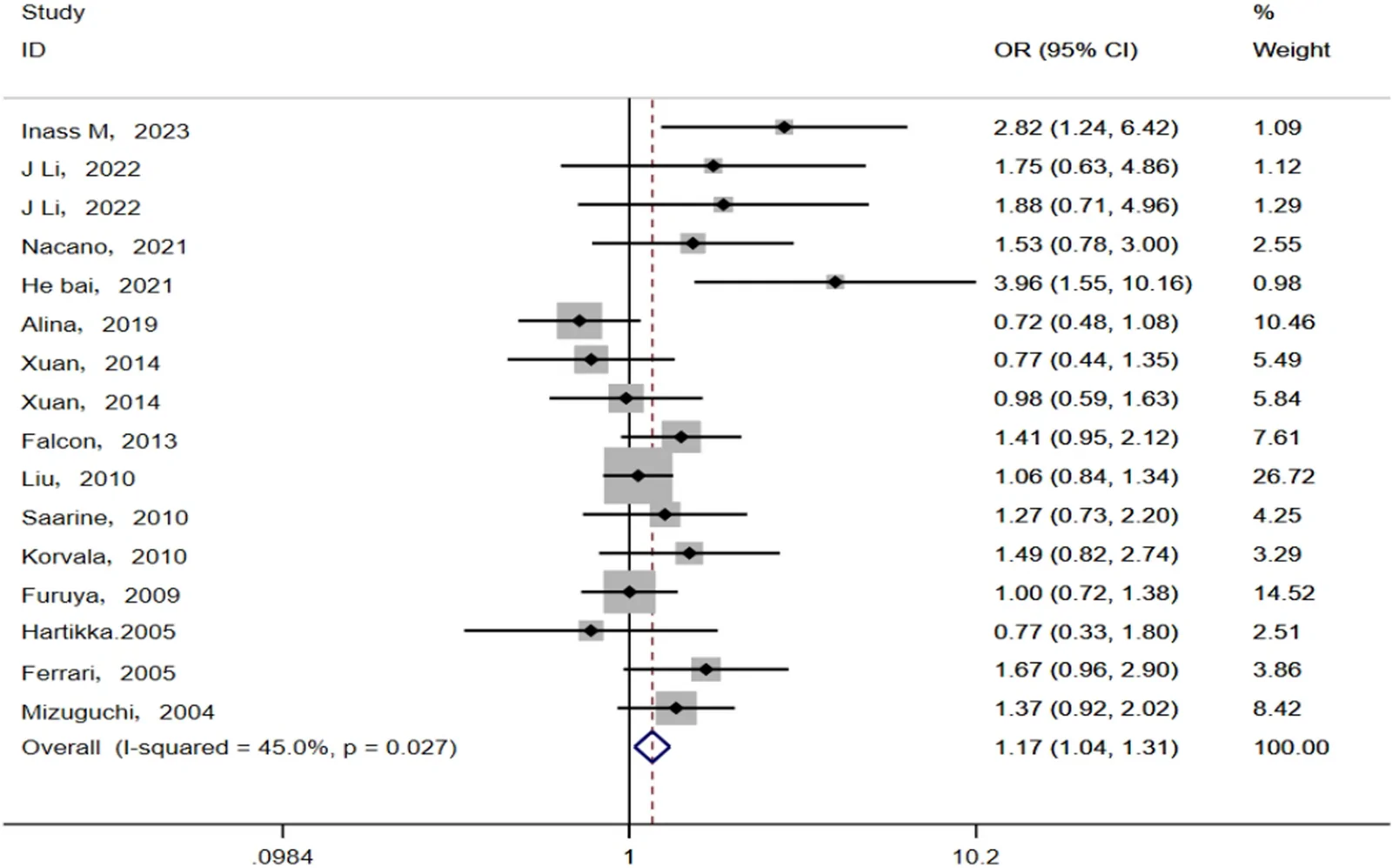
The forest plots on the association between *LRP5* rs3736228 polymorphism and osteoporosis risk under the allele model

Using five genetic models(allelic, homozygous, heterozygous, dominant, and recessive), the association of the *LRP5* rs3736228 polymorphism with osteoporosis and fracture risk was assessed. The meta-analysis revealed a significant correlation between the *LRP5* rs3736228.

polymorphism and increased risk of osteoporosis in 4 genetic models: allelic [OR = 1.17, 95%CI(1.04–1.31), *P* = 0.011], homozygous [OR = 1.47, 95%CI(1.07–2.01), *P =* 0.018], dominant [OR = 1.44, 95%CI(1.05–2.97), *P =* 0.023], and recessive [OR = 1.16, 95%CI(1.01–1.33), *P* = 0.041]. However, the heterozygous model for the *LRP5* rs3736228 polymorphism [OR = 1.34, 95%CI(0.96–1.88), *P* = 0.09]did not show a statistically significant difference between cases and controls in the 14 included studies. The summarized outcomes of the combined analysis are presented in Fig. 2 and Table 4.

### Heterogeneity analyses and sensitivity analyses

During the statistical analyses, various potential sources of heterogeneity, including the publication year, sex, ethnicity, source of controls, illnesses, genotyping technique, and HWE, were investigated. Subsequently, meta-regression analysis was employed to identify the sources of variation, as shown in Table 3. Univariate analysis did not yield any statistically significant moderators(*P* < 0.05), indicating that no single covariate alone adequately explained the observed heterogeneity. In contrast, multivariate meta-regression, which evaluates all covariates simultaneously, identified three significant moderators: publication year(Coefficient = 0.047, *P* = 0.026), source of controls(Coefficient = 0.996, *P* = 0.012), and disease type(Coefficient = 0.442, *P* = 0.033). Specifically, more recent studies, those employing hospital-based controls, and those focusing on osteoporosis rather than fracture were associated with larger effect size estimates.

**Table 3:** Univariate and multivariate meta regression analyses of potential source of heterogeneity under the allele model

Heterogeneity factors	Coefficient	SE	Z	*P*	LL	UL
Publication year						
Univariate	0.017	0.017	1.01	0.331	-0.019	0.053
Multivariate	0.047	0.017	2.71	0.026	0.007	0.087
Ethnicity						
Univariate	0.041	0.205	0.20	0.846	-0.399	0.480
Multivariate	-0.391	0.224	-1.74	0.119	-0.907	0.126
Source of controls						
Univariate	0.187	0.211	0.89	0.390	-0.265	0.640
Multivariate	0.996	0.310	3.21	0.012	0.280	1.712
Disease						
Univariate	0.123	0.211	0.58	0.570	-0.330	0.577
Multivariate	0.442	0.171	2.58	0.033	0.047	0.838
Genotyping method						
Univariate	-0.174	0.200	-0.87	0.400	-0.604	0.256
Multivariate	-0.312	0.267	-1.17	0.275	-0.927	0.302
Sex						
Univariate	0.177	0.137	1.29	0.219	-0.118	0.470
Multivariate	0.126	0.218	0.86	0.415	-0.316	0.691
Deviation from HWE(Yes vs. No)						
Univariate	-0.072	0.263	-0.27	0.789	-0.637	0.493
Multivariate	0.201	0.255	0.79	0.454	-0.388	0.790

For the variable 'Deviation from HWE, 'the analysis was performed with studies exhibiting deviation (Yes) as the reference group

**Fig. 3:**
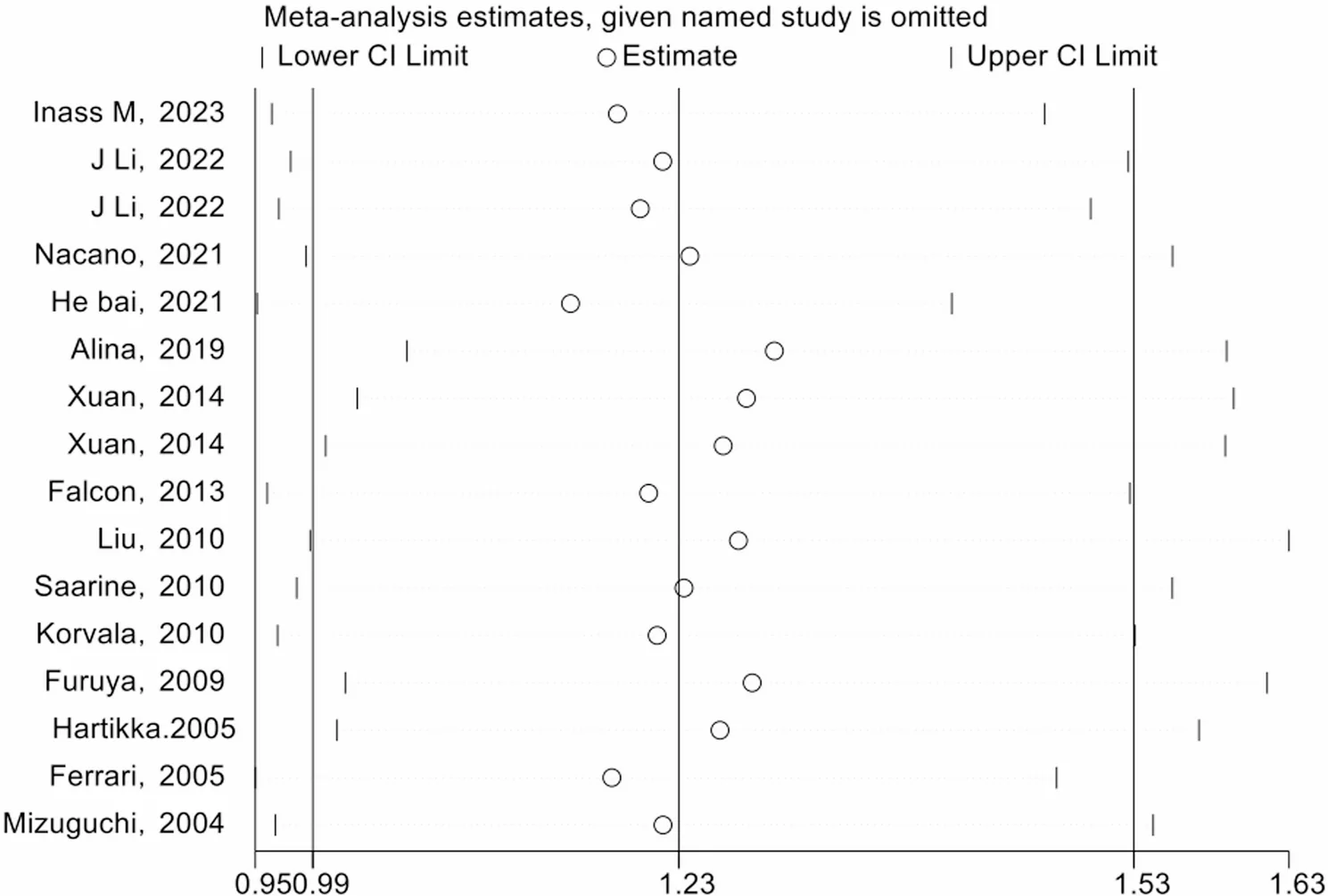
Sensitivity analysis for the relationships between *LRP5* rs3736228 polymorphism under the allele model

In order to enhance the reliability of the meta-analysis, a sensitivity analysis was conducted, systematically excluding each study one at a time. This analysis aimed to assess whether the exclusion of any single study significantly influenced the overall findings. The results of the sensitivity analysis indicated that none of the studies included in the current meta-analysis had a substantial impact on the pooled OR (Fig. 3).

### Publication bias

**Fig. 4:**
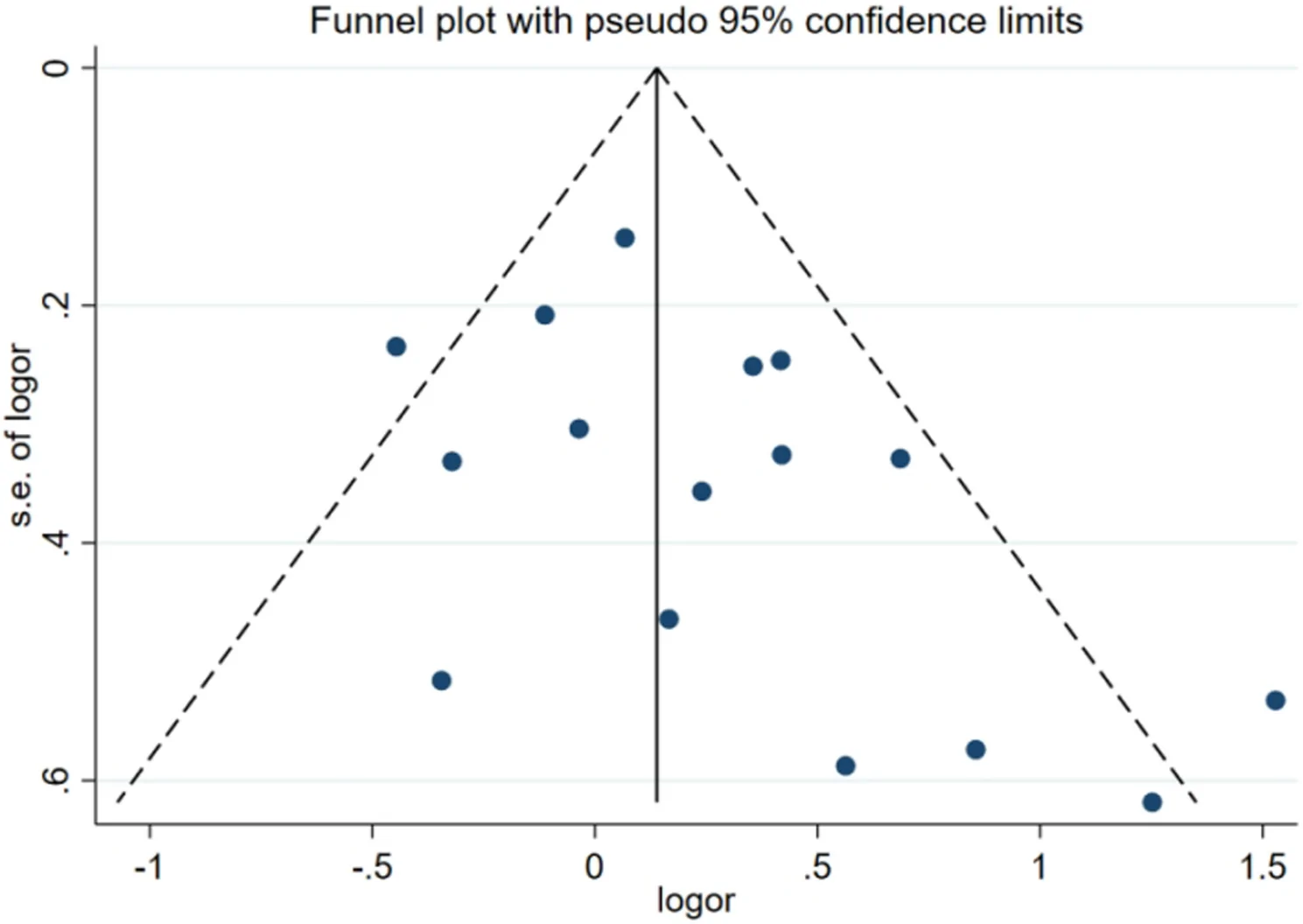
Publication bias with *LRP5* rs3736228 polymorphism with confidence limits under the allele model

In order to delve deeper into publication bias, a funnel plot and Begg’s test were employed. In both the overall and broken-down analysis(*P* > 0.05 in all genetic models), Begg’s test failed to identify any evidence of an asymmetric distribution in the funnel plots, as shown in Fig. 4 and Table 4. This suggested that no publication bias was present in any of the studies. Egger’s test indicated a slight publication bias in the allele model(t = V2.2,*P* *= 0.045*); however, analysis of the remaining subgroups showed no publication bias (Table 4).

**Table 4:** Meta-analysis of the association of LRP5 rs3736228 polymorphism with risk of osteoporosis

Polymorphism	Subgroup	Num.of.studies	sample size	Test of association			Test of Heterogenity				Begg test for publications		Egger test for publications	
				OR	95%CI	P-value	model	Q	P-value	I^2^, %	z	P-value	t	P-value
T vs. C	overall	16	1549/3345	1.17	1.04–1.31	0.011	F	27.28	0.027	45.00%	1.4	0.163	2.2	0.045
	Caucasian	7	648/1593	1.28	0.93–1.77	0.134	R	13.81	0.032	56.60%	0.3	0.764	0.93	0.397
	Asian	9	901/1752	1.14	0.99–1.32	0.07	F	13.14	0.107	39.10%	1.15	0.251	2.01	0.085
	osteoporosis	12	936/1701	1.32	1.01–1.71	0.039	R	24.59	0.01	55.30%	1.17	0.244	1.65	0.13
	fracture	4	613/1644	1.09	0.92–1.29	0.324	F	1.66	0.646	0.00%	1.02	0.308	2.18	0.161
	Taqman	8	911/2457	1.10	0.96–1.27	0.164	F	13.91	0.053	49.70%	0.12	0.902	0.92	0.392
	Non-Taqman	8	638/888	1.34	1.07–1.67	0.01	F	11.74	0.11	40.40%	1.11	0.266	2.2	0.07
	population-based	10	971/2882	1.21	1.06–1.39	0.005	F	10.87	0.284	17.20%	1.43	0.152	2.08	0.071
	Hospital-based	6	578/463	1.21	0.76–1.92	0.419	R	14.4	0.013	65.30%	1.13	0.26	3.83	0.019
	Con-HWE	13	1168/2874	1.18	1.04–1.34	0.010	F	15.17	0.232	20.90%	1.4	0.161	1.75	0.108
	Non-HWE	3	381/471	1.39	0.61–3.17	0.434	R	11.49	0.003	82.60%	1.04	0.296	9.59	0.066
	female	11	1154/2596	1.14	0.95–1.38	0.135	R	16.92	0.076	40.90%	0.62	0.533	1.56	0.153
	male	2	222/326	1.59	1.06–2.39	0.026	F	0.07	0.791	0.00%	0	1	NA	NA
	mix	3	173/423	1.51	0.67–3.42	0.065	R	6.72	0.035	70.20%	0	1	0.42	0.748
	premenopause	1	12/591	2.82	1.24–6.42	0.014								
	postmenopause	10	1142/2005	1.08	0.95–1.23	0.242	F	11.84	0.222	24.00%	0.18	0.858	0.96	0.365
TT VS CC	overall	16	1549/3345	1.47	1.07–2.01	0.018	F	9.04	0.876	0	1.31	0.192	1.28	0.221
	Caucasian	7	648/1593	1.6	0.89–2.89	0.116	F	3.93	0.686	0	0.3	0.764	0.36	0.736
	Asian	9	901/1752	1.41	0.97–2.06	0.072	F	4.84	0.774	0	0.73	0.466	1.02	0.34
	osteoporosis	12	936/1701	1.86	1.13–3.06	0.015	F	6.76	0.818	0	0.07	0.945	-0.43	0.679
	fracture	4	613/1644	1.23	0.81–1.88	0.328	F	0.2	0.978	0	1.02	0.308	1.37	0.304
	Taqman	8	911/2457	1.53	1.05–2.21	0.026	F	7.62	0.367	8.10%	0.87	0.386	1.57	0.167
	Non-Taqman	8	638/888	1.32	0.72–2.43	0.374	F	1.15	0.992	0	1.61	0.108	1.12	0.306
	population-based	10	971/2882	1.49	1.06–2.10	0.021	F	7.79	0.555	0	1.07	0.283	1.57	0.155
	Hospital-based	6	578/463	1.32	0.55–3.17	0.537	F	1.12	0.952	0	1.5	0.133	3.61	0.023
	Con-HWE	13	1168/2874	1.47	1.04–2.08	0.03	F	8.72	0.727	0	0.79	0.428	1.02	0.33
	Non-HWE	3	381/471	1.46	0.66–2.30	0.351	F	0.32	0.854	0	1.04	0.296	1.59	0.358
	female	11	1154/2596	1.5	1.05–2.14	0.027	F	8.33	0.597	0	1.09	0.276	1.32	0.218
	male	2	222/326	1.52	0.30–7.71	0.612	F	0.01	0.904	0	0	1	NA	NA
	mix	3	173/423	1.32	0.60–2.88	0.489	F	0.57	0.753	0	0	1	0.15	0.904
	menopause	1	12/591	7.83	1.37–44.93	0.021								
	Non-menopause	10	1142/2005	1.43	1.00-2.06	0.049	F	4.85	0.847	0	0.54	0.592	0.95	0.372
TT VS CT	overall	16	1549/3345	1.34	0.96–1.88	0.09	F	7.24	0.951	0	0.32	0.753	-0.07	0.948
	Caucasian	7	648/1593	1.33	0.68–2.58	0.403	F	1.53	0.958	0	0	1	0.13	0.903
	Asian	9	901/1752	1.34	0.91–1.99	0.14	F	5.71	0.679	0	0.1	0.917	-0.09	0.934
	osteoporosis	12	936/1701	1.49	0.89–2.47	0.126	F	6.07	0.869	0	1.3	0.193	-0.93	0.374
	fracture	4	613/1644	1.23	0.78–1.94	0.376	F	0.8	0.848	0	0.34	0.734	-0.15	0.892
	Taqman	8	911/2457	1.49	1.01–2.18	0.043	F	4.98	0.662	0	0.37	0.711	1.55	0.173
	Non-Taqman	8	638/888	0.95	0.47–1.94	0.894	F	0.91	0.996	0	0.62	0.536	-0.41	0.697
	population-based	10	971/2882	1.38	0.96–1.98	0.084	F	5.57	0.782	0	0	1	0.53	0.613
	Hospital-based	6	578/463	1.13	0.45–2.82	0.791	F	1.41	0.924	0	0	1	-0.11	0.92
	Con-HWE	13	1168/2874	1.37	0.95–1.96	0.09	F	6.31	0.9	0	0.43	0.669	0.06	0.95
	Non-HWE	3	381/471	1.17	0.43–3.19	0.763	F	0.8	0.672	0	0	1	0.04	0.977
	female	11	1154/2596	1.43	0.99–2.07	0.054	F	5.99	0.816	0	0	1	0.7	0.499
	male	2	222/326	0.83	0.16–4.32	0.824	F	0.08	0.776	0	0	1	NA	NA
	mix	3	173/423	0.96	0.34–2.70	0.944	F	0.22	0.895	0	1.04	0.296	-0.86	0.549
	menopause	1	12/591	2.65	0.51–13.89	0.248								
	Non-menopause	10	1142/2005	1.40	0.96–2.04	0.079	F	5.46	0.792	0	0	1	0.49	0.635
TT VS CC + CT	overall	16	1549/3345	1.44	1.05–2.97	0.023	F	7.75	0.933	0	0.77	0.444	0.97	0.349
	Caucasian	7	648/1593	1.52	0.85–2.72	0.162	F	2.56	0.861	0	0	1	0.13	0.9
	Asian	9	901/1752	1.41	0.97–2.04	0.072	F	5.09	0.748	0	0.94	0.348	0.77	0.467
	osteoporosis	12	936/1701	1.74	1.07–2.84	0.027	F	5.93	0.878	0	1.03	0.304	-0.98	0.35
	fracture	4	613/1644	1.25	0.83–2.19	0.291	F	0.36	0.948	0	0.34	0.734	0.66	0.575
	Taqman	8	911/2457	1.51	1.05–2.18	0.026	F	6.49	0.484	0	0.87	0.386	1.5	0.184
	Non-Taqman	8	638/888	1.25	0.68–2.31	0.469	F	0.85	0.997	0	1.11	0.266	0.81	0.447
	population-based	10	971/2882	1.46	1.04–2.04	0.027	F	6.75	0.663	0.00%	0.54	0.592	1.23	0.253
	Hospital-based	6	578/463	1.30	0.54–3.13	0.552	F	0.89	0.971	0.00%	1.13	0.26	2.5	0.067
	Con-HWE	13	1168/2874	1.44	1.02–2.02	0.038	F	7.50	0.823	0.00%	0.18	0.855	0.69	0.503
	Non-HWE	3	381/471	1.44	0.66–3.14	0.365	F	0.26	0.879	0.00%	1.04	0.296	3.95	0.158
	female	11	1154/2596	1.48	1.04–2.09	0.03	F	7.17	0.709	0	0.47	0.64	1.09	0.305
	male	2	222/326	1.27	0.25–6.37	0.774	F	0.06	0.812	0	0	1	NA	NA
	mix	3	173/423	1.3	0.60–2.84	0.511	F	0.35	0.839	0	0	1	0.21	0.869
	menopause	1	12/591	4.72	0.98–22.76	0.053								
	Non-menopause	10	1142/2005	1.42	0.99–2.03	0.054	F	5.06	0.829	0	0.54	0.592	0.78	0.461
TT + CT vs. CC	overall	16	1549/3345	1.16	1.01–1.33	0.041	F	28.83	0.017	0.48	1.58	0.115	2.03	0.062
	Caucasian	7	648/1593	1.3	0.87–1.93	0.205	R	15.02	0.02	0.601	0.6	0.548	0.99	0.368
	Asian	9	901/1752	1.13	0.95–1.35	0.175	F	13.52	0.095	0.408	1.15	0.251	1.7	0.133
	osteoporosis	12	936/1701	1.33	0.97–1.84	0.08	R	25.95	0.007	0.576	0.75	0.451	1.08	0.306
	fracture	4	613/1644	1.07	0.87–1.32	0.499	F	2.15	0.542	0	0.34	0.734	1	0.423
	Taqman	8	911/2457	1.06	0.90–1.26	0.483	F	13.22	0.067	0.47	0.87	0.386	0.66	0.532
	Non-Taqman	8	638/888	1.42	1.09–1.83	0.008	F	0.08	0.08	0.449	0.87	0.386	1.94	0.1
	population-based	10	971/2882	1.22	1.03–1.43	0.017	F	10.94	0.28	17.80%	0.72	0.474	1.49	0.175
	Hospital-based	6	578/463	1.24	0.71–2.16	0.444	R	15.95	0.007	68.70%	1.13	0.26	3.82	0.019
	Con-HWE	13	1168/2874	1.19	1.02–1.38	0.029	F	15.55	0.213	22.80%	1.4	0.161	1.75	0.108
	Non-HWE	3	381/471	1.42	0.52–3.89	0.499	R	12.35	0.002	83.80%	1.04	0.296	12.59	0.05
	female	11	1154/2596	1.07	0.92–1.25	0.392	F	16.68	0.082	40.00%	1.4	0.161	1.45	0.181
	male	2	222/326	1.74	1.11–2.73	0.016	F	0.33	0.565	0.00%	0	1	NA	NA
	mix	3	173/423	1.57	0.60–4.15	0.361	R	6.81	0.033	70.60%	0	1	0.32	0.801
	menopause	**1**	12/591	3.50	1.04–11.75	0.043								
	Non-menopause	**10**	1142/2005	1.06	0.86–1.30	0.606	F	12.92	0.166	30.40%	0.72	0.474	0.84	0.426

mix: studies enrolling both male and female participants

### Subgroup analyses

The primary findings of this updated meta-analysis are listed in Table 4. The combined ORs were calculated for the allelic, dominant, heterozygous, recessive, and homozygous models. When stratified by ethnicity, no statistically significant difference was observed between Caucasians and Asians under any model (all *P* > 0.05, Table 4). The analysis by control source indicated that rs3736228 polymorphism was associated with increased vulnerability to osteoporosis and fractures in allelic, homozygous, dominant, and recessive models [T vs. C: OR = 1.21, 95%CI(1.06∼1.39), *P* = 0.005; TT vs. CC: OR = 1.49, 95%CI(1.06–2.10), *P* = 0.021; TT + TC vs. CC: OR = 1.22, 95%CI(1.03–1.43), *P* = 0.017;TT vs. CC + CT: OR = 1.46, 95%CI(1.04–2.04), *P* = 0.027]. However, no strong association was found in the hospital subgroup across all genetic models(*P* > 0.05). In a disease-based subgroup, a higher risk of developing osteoporosis was observed under the allele, homozygous, and dominant models [T vs. C: OR = 1.32, 95%CI(1.01–1.71), *P* = 0.039; TT vs. CC: OR = 1.86, 95%CI(1.03–3.06), *P =* 0.015;TT + TC vs. CC: OR = 1.74, 95%CI(1.07–2.84), *P =* 0.027). However, fractures did not show comparable outcomes(all *P* > 0.05). Stratifying by genetic methods, the carriers of the rs3736228 polymorphism in the TaqMan assay subgroups, particularly in homozygous, heterozygous, and recessive models, were associated with increased susceptibility to osteoporosis and fracture(*P* < 0.05). In the non-TaqMan assay subgroups, significant associations were observed in the allelic and dominant model(*P* < 0.05).

Subgroup analysis with HWE stratification demonstrated a close association between the rs3736228 polymorphism of the *LRP5* gene and the risk of osteoporosis andfracture in four genetic models (Table 4): allelic model [T vs. C: OR = 1.18, 95%CI(1.03–1.34), *P =* 0.010]; homozygous model [TT vs. CC: OR = 1.47, 95%CI(1.04–2.08, *P =* 0.03]; dominant model [TT + CT vs. CC: OR = 1.19; 95%CI(1.02–1.38), *P* = 0.029]; and recessive model [TT vs. CC + CT: OR = 1.44; 95%CI(1.02–2.02), *P* = 0.038]but not in the heterozygous model(*P* > 0.05). Among the child subgroup, the analysis indicated that the rs3736228 polymorphism of the *LRP5* gene marginally increased the risk of osteoporosis and fracture in the allelic model [T vs. C OR = 1.82; 95%CI(1.05–3.14), *P* = 0.033]and recessive model [TT vs. CC + CT OR = 2.06; 95%CI(1.09–3.90), *P* = 0.026].

In terms of sex subgroups, the LRP5 gene rs3736228 polymorphism increased the risk of osteoporosis in women in a homozygous model [TT vs. CC: OR = 1.5, 95%CI(1.05–2.14), *P* = 0.027]and recessive model [TT vs. CC + CT: OR = 1.48, 95%CI(1.04–2.09), *P* = 0.03]but not in the allele model [T vs. C: OR = 1.59, 95%CI(1.06–2.39), *P* = 0.026]and dominant model [TT + CT vs. CC: OR = 1.74, 95%CI(1.11–2.73), *P* = 0.016]. Similarly, in the female menopausal subgroup, the LRP5 rs3736228 polymorphism increased the risk of osteoporosis in women who were not postmenopausal in the allelic model [T vs. C: OR = 2.82, 95%CI(1.24–6.42), *P* = 0.014], homozygous model [TT vs. CC: OR = 1.43, 95%CI (1.00-2.06), *P* = 0.049], and recessive model [TT vs. CC + CT: OR = 3.50, 95%CI(1.04–11.75), *P* = 0.043], as listed in the Table 4. However, the sample size was limited to one research, which might have limited the statistical power of this finding. Therefore, the current meta-analysis did not consider it sufficient to establish a significant link, even though there was a substantial association in the community of postmenopausal women.

### Confidence in identified genetic associations

The genetic connections were classified as"positive results"when the following conditions were satisfied: (1)*r* < 0.05 for two or more genetic models; (2)FPRP < 0.2;(3)statistical power > 0.8; and(4)*I2* < 50%. If the following conditions were satisfied, a connection was regarded as a"less credible positive result”:(1)*P* < 0.05 in a minimum of one genetic model; (2)FPRP > 0.2,*I*2 > 50%,or their statistical power ranged from 50%to 79%. The associations with *P* > 0.05 were categorized as"zero"or"negative”. Following credibility assessment,“less-credible positive results”were discovered for the *LRP5* rs3736228 allele, recessive, dominant model, and homozygous models in the overall analysis. The findings of thecurrent meta-analysis’s credibility assessment are listed in Table 5.

**Table 5:** False positive report probability values for the current meta-analysis

Variable	OR(95%CI)	I2(%)	Ph	Statistical power		Prior probability of 0.001	
				OR = 1.2	OR = 1.5	OR = 1.2	OR = 1.5
*LRP5* rs3736228							
T vs. C(allele model)	1.17(1.04–1.31)	45.00%	0.011	0.670	1.000	0.906	0.866
TT VS CC(homozygotes model)	1.47(1.07–2.01)	0.00%	0.018	0.102	0.550	0.994	0.966
TT VS CT(heterozygous model)	1.34(0.96–1.88)	0.00%	0.09	0.261	0.743	0.997	0.992
TT VS CC + CT(recessive model)	1.44(1.05–2.97)	0.00%	0.041	0.311	0.544	0.999	0.998
TT + CT vs. CC(dominant model)	1.16(1.01–1.33)	48.0%	0.023	0.686	1.000	0.980	0.971

## Discussion

The LRP5 rs3736228 (Ala1330Val) polymorphism has been firmly established as a genome-wide significant locus for bone mineral density (BMD) and fracture risk through multiple large-scale genome-wide association studies (GWAS) and consortium-level meta-analyses [18, 32]. *LRP5*, an integral component of the low-density lipoprotein(LDL)receptor family, plays a crucial role in skeletal homeostasis, engaging in ligand binding and internalization through receptor-mediated endocytosis [43]. The normal morphology and developmental processes are intricately linked to the *LRP5* signaling system, while the pathophysiology and osteogenesis of osteoporosis are significantly influenced by the Wnt signaling pathway.

In terms of biological mechanisms, functional investigations [44–46] are now framed as potential explanations for the significant associations we observed. Specifically, we state that the significant association of the *LRP5* rs3736228 polymorphism with osteoporosis risk, particularly its differential effects across subgroups(e.g.,sex, disease phenotype), may be biologically plausible given its role in the Wnt signaling pathway and its potential *cis*-regulatory effects on genes like *CPT1A* and *MTL5*, which are involved in bone metabolism and cell differentiation. The reference to GWAS enrichment in the Wnt pathway further reinforces that our genetic finding aligns with a major known biological pathway for osteoporosis [47].

To complement the existing GWAS evidence and address these granularity gaps, the present updated meta-analysis synthesized data from 14 studies encompassing 4,894 participants, providing updated pooled evidence that the *LRP5* rs3736228 polymorphism is significantly associated with an increased risk of osteoporosis and related fractures. This conclusion is supported by significant odds ratios across multiple genetic models, including the allelic, homozygous, dominant, and recessive models.Collectively, these results add an updated layer of evidence to the existing literature by providing a quantitative synthesis with a larger, more comprehensive dataset.Our findings do not replace GWAS evidence but rather extend it by offering clinically actionable, subgroup-specific risk estimates that may aid in refining risk stratification in diverse patient populations.

The data highlighted significant variations in the relative distribution of the A1330V polymorphism among different populations, with CC genotypes observed in 68%of Chinese females, 46%of Japanese females, 73%of Dutch females, and 65.9% of Maya-Mestizo females [18].Xu GY [20] suggested a potential connection between the rs3736228 polymorphism and osteoporosis and bone fracture risks in Caucasians but not in Asians. According to Van Meurs et al. [48], typical genetic variations in the *LRP5* gene might be reliably connected to fracture risk and BMD in various white groups. Contrary to previous studies, our subgroup results revealed no statistically significant difference between Caucasians and Asians. Additionally, the disease-type stratification study indicated that the rs3736228 mutation might be associated with the onset of osteoporosis; however, its predictive value for the etiology of fractures remains uncertain.

Males, often overlooked in osteoporosis discussions, have demonstrated vulnerability, with prevalence rates of 18%for hip osteopenia and 2%for osteoporosis [49]. Ferrari et al. [41] suggested that *LRP5* gene variation could be a hereditary factor influencing men’s susceptibility to idiopathic osteoporosis. According to the current meta-analysis, the *LRP5* rs3736228 polymorphism might increase the risk of osteoporosis in men. Among females, including those premenopausal, the *LRP5* rs3736228 polymorphism increased the risk of osteoporosis and fractures. Within the age subgroup, a recent large cohort study involving 372 patients with early-onset osteoporosis revealed rare *LRP5* variants in 8.3% of the patients [15]. The stratified analyses yielded several insightful findings. First, the association appeared more pronounced in osteoporosis patients than in fracture-only cohorts. This suggests that rs3736228 may exert a stronger influence on the pathological process of bone density loss itself, while the clinical outcome of a fracture is modulated by additional non-genetic factors like fall risk. Second, we observed distinct genetic model associations between genders. The risk in males was significant in the allele and dominant models, whereas in females, it was evident in the homozygous and recessive models. This sexual dimorphism may reflect complex interactions between the *LRP5* pathway and sex hormones, warranting further functional investigation. This observed sexual dimorphism in genetic association patterns suggests a potential differential biological role or interaction of the *LRP5* pathway in males and females. While the precise mechanism remains to be fully elucidated, this observation is consistent with the known interplay between sex hormones(e.g.,estrogen)and the Wnt/β-catenin signaling pathway in regulating bone metabolism [46]. Future functional studies are warranted to investigate whether the effect of the *LRP5* rs3736228 polymorphism is modulated by sex hormone status. Finally, the significant association observed in the premenopausal female subgroup, provides preliminary evidence suggesting that genetic susceptibility to osteoporosis may not be confined to postmenopausal women, a notion that warrants further investigation in larger, well-designed cohorts.

Meta-regression analysis was employed to investigate the origins of heterogeneity, which revealed that the factors contributing to heterogeneity were the type of illness, the publication year, and the source of control. Furthermore, in contemporary meta-analyses, the utilization of various genetic models necessitated multiple comparisons, requiring adjustment of the pooled *P*-value. Key markers among the Venice criteria include statistical power and *I*2. Therefore, the confidence in statistically significant connections was evaluated using the Venice criterion [29] and FPRP [30]. Ultimately, the homozygous, recessive, dominant, and C > T allele models of *LRP5* rs3736228 showed“less”credible positive results. This honest appraisal underscores the need for even larger, well-designed primary studies to solidify these associations.

Moreover, studies with small sample sizes are more prone to biases, random errors, and false positive outcomes in molecular epidemiology investigations [30]. The allele model showed a minimal bias in the Egger test results. However, Begg’s funnel plot was used to demonstrate the basic symmetry of each model. The following benefits of this meta-analysis might be emphasized: (1)application of the Venice criterion and FPRP for credibility assessment; (2)evaluation of the quality of relevant research, (3)a more comprehensive inclusion of studies with more precise and detailed data compared to earlier meta-analysis; and(4)thorough subgroup analyses with a noteworthy division of women based not only on gender but also on menopausal status [50].

The current study has some limitations. First, while the current study underwent thorough bias diagnostics and revealed no overt bias, the possibility of publication bias cannot be completely ruled out. Furthermore, the inclusion of some primary studies that did not conform to Hardy-Weinberg equilibrium(HWE)may influence the pooled effect estimates. Certain subgroup analyses(e.g.menopause)were also constrained by small sample sizes, potentially limiting statistical power, and the potential for overlapping cohorts across original studies may have inflated the significance of some associations. Third, the findings were limited to Asians and Caucasians since these two ethnic groups were the sole focus of the demographic regional subgroup analysis, limiting generalizability to other ethnic groups.Fourth, our meta-analysis was restricted to a single candidate locus, which cannot reflect the polygenic architecture underlying bone fragility. Consequently, compared with previously published large-scale genome-wide association studies (GWAS), the incremental evidence provided by the present study is modest.Lastly, while hereditary factors are a focus, this study did not account for numerous non-genetic factors known to influence osteoporosis risk, including physical activity, lactation, estrogen replacement treatment, corticosteroids, smoking, alcohol use, and calcium supplementation [51]. The inability to adjust for these potential confounders means that the magnitude and even the direction of the identified genetic associations could be biased, limiting the clinical interpretability of the findings in isolation from lifestyle and medical contexts.

## Conclusion

This updated meta-analysis demonstrates that the *LRP5* rs3736228 polymorphism increases the risk of osteoporosis and fracture, supporting results from large-scale GWAS.Our main contribution is to provide subgroup-specific estimates, stratified by sex, menopausal status, and ethnicity, to supplement GWAS findings at the population level.The sex-specific genetic models and suggestive connection in premenopausal women and children emphasize understudied categories that deserve focus in future studies.

However, as indicated by the“less” credible positive’assessment in our credibility analysis, these findings should be interpreted with caution. Instead, our results underscore the necessity for larger, well-designed prospective studies with sufficient statistical power to confirm these associations. This work highlights critical subgroups(such as males and premenopausal females)and specific genetic models that warrant priority in future investigations. Ultimately, clarifying the role of *LRP5* and its interaction with other factors is essential for understanding the genetic architecture of osteoporosis and may inform the development of more comprehensive risk assessment tools in the long term.

## Supplementary Information

Supplementary Material 1.

Supplementary Material 2.

Supplementary Material 3.

Supplementary Material 4.

Supplementary Material 5.

## Data Availability

The data and materials supporting the findings of this study are available in supplementary files.
