# Supplementary material for: Association of rs3736228 polymorphism in LRP5 gene with bone fracture and osteoporosis: a systematic review and updated meta-analysis
Source: BMC Musculoskelet Disord. 2026 Jul 27;27:898. doi: 10.1186/s12891-026-10270-9 (PMC13689123; doi:10.1186/s12891-026-10270-9)
Supplement: Supplementary file 2 [file 12891_2026_10270_MOESM2_ESM.docx]

**Supplementary Table .** Retrieval steps and results in PubMed (The retrieval time: From inception to November.1.2023).

| Search | Query | Items found |
| --- | --- | --- |
| #1 | osteoporosis[MeSH terms] | 63370 |
| #2 | fractures[MeSH Terms] | 210268 |
| #3 | "osteoporosis"[Title/Abstract] OR ("age-related"[All Fields] AND "OP"[Title/Abstract]) OR "age related bone loss"[Title/Abstract] OR "OP"[Title/Abstract] OR (("juvenile"[All Fields] OR "juvenile s"[All Fields] OR "juveniles"[All Fields] OR "juvenility"[All Fields]) AND "OP"[Title/Abstract]) OR "bone fracture"[Title/Abstract] OR "fractures"[Title/Abstract] OR "broken bone"[Title/Abstract] | 276638 |
| #4 | #1 OR #2 OR #3 | 366638 |
| #5 | "low density lipoprotein receptor related protein 5"[MeSH Terms] | 746 |
| #6 | "low density lipoprotein receptor related protein 5"[Title/Abstract] OR "LRP5"[Title/Abstract] | 1515 |
| #7 | #5 OR #6 | 1616 |
| #8 | Polymorphism [MeSH terms] | 304426 |
| #9 | "Polymorphism"[Title/Abstract] OR "single nucleotide mutation"[Title/Abstract] OR "mutation"[Title/Abstract] OR "genetic variation"[Title/Abstract] OR "variation"[Title/Abstract] | 1159295 |
| #10 | #8 OR #9 | 1287635 |
| #11 | #4 AND #7 AND #9 | 68 |

Retrieval steps and results in web of science (The retrieval time: From inception to November.1.2023).

| Search | Query | Items found |
| --- | --- | --- |
| #1 | 1: ((((((((TS=(osteoporosis)) OR TS=(Age-Related OP)) OR TS=(Age-Related Bone Loss)) AND TS=(OP)) OR TS=(Juvenile OP)) OR TS=(Juvenile OP)) OR TS=(Bone Fracture)) OR TS=(Broken Bone)) OR TS=(Fractures) | 1355558 |
| #2 | (((TS=(LRP5)) OR TS=(low density lipoprotein receptor related protein 5)) OR TS=(low density lipoprotein receptor related protein 5)) OR TS=(low density lipoprotein receptor related protein 5) | 7366 |
| #3 | 3: #2 AND #1 | 457 |
| #4 | 4: ((((TS=(polymorphism)) OR TS=(genetic polymorphism)) OR TS=(single nucleotide  polymorphism)) OR TS=(variation)) OR TS=(genetic variation) | 4597172 |
| #5 | 5: #3 AND #4 | 195 |
