# Supplementary material for: Association of rs3736228 polymorphism in LRP5 gene with bone fracture and osteoporosis: a systematic review and updated meta-analysis
Source: BMC Musculoskelet Disord. 2026 Jul 27;27:898. doi: 10.1186/s12891-026-10270-9 (PMC13689123; doi:10.1186/s12891-026-10270-9)
Supplement: Supplementary file 3 [file 12891_2026_10270_MOESM3_ESM.docx]

| Certanity of assessment | | | | | | | | Certanity | Importance |
| --- | --- | --- | --- | --- | --- | --- | --- | --- | --- |
| **N_2_ of studies** | **Study design** | **Risk of bias** | **Incosistency** | **Indirectness** | **Imprecision** | **Other consideration** | |  |  |
| **LRP5 rs3736228 polymorphism --Osteoporosis and frature** | | | | | | | | | |
| **14** | **Case-control studies** | **Not serious** | **Not serious** | **Not serious** | **Not serious** | | **All plausible residual confuding would reduce demostrated the effect** | **⊕⊕○○** | **IMPORTANT** |

**GRADE Working Group grades of evidence**

**High Certainty:**The authors have a lot of confidence that the true effect is similar to the estimated effect

**Moderate Certainty:**The authors believe that the true effect is probably close to the estimated effect

**Low Certainty:**The true effect might be markedly different from the estimated effec**t**

**Very low certainty:**The true effect is probably markedly different from the estimated effect
