# Supplementary material for: Association of rs3736228 polymorphism in LRP5 gene with bone fracture and osteoporosis: a systematic review and updated meta-analysis
Source: BMC Musculoskelet Disord. 2026 Jul 27;27:898. doi: 10.1186/s12891-026-10270-9 (PMC13689123; doi:10.1186/s12891-026-10270-9)
Supplement: Supplementary file 4 [file 12891_2026_10270_MOESM5_ESM.docx]

**TableS1:Results of quality assessment using the Newscastle-ottawa scale for case-control studies**

| **Study** | **Selection** | | | | **Comparability**  **Control for important factor** | **Exposure** | | | **Scores** |
| --- | --- | --- | --- | --- | --- | --- | --- | --- | --- |
|  | **Adequate**  **Definitions**  **Of**  **cases** | **Representativeness**  **Of the cases** | **Selections**  **Of**  **controls** | **Definitions**  **Of**  **Controls** |  | **Ascertainment of exposure** | **Same method of ascertainment for cases and controls** | **Non response rate** |  |
| **J Li**  **2022** | **✭** | **✭** |  | **✭** | **✭✭** | **✭** | **✭** | **✭** | **8** |
| **Masaki**  **2021** | **✭** | **✭** | **✭** | **✭** |  | **✭** | **✭** | **✭** | **7** |
| **He bai**  **2021** | **✭** | **✭** |  | **✭** | **✭✭** | **✭** | **✭** | **✭** | **8** |
| **Alina**  **2019** | **✭** | **✭** |  | **✭** | **✭✭** | **✭** | **✭** | **✭** | **8** |
| **Xuan**  **2014** | **✭** | **✭** |  | **✭** | **✭✭** | **✭** | **✭** | **✭** | **8** |
| **Falcon**  **2013** | **✭** | **✭** | **✭** | **✭** | **✭** | **✭** | **✭** | **✭** | **8** |
| **Korvala**  **2010** | **✭** | **✭** | **✭** | **✭** | **✭** | **✭** | **✭** |  | **7** |
| **Saarine**  **2010** | **✭** | **✭** | **✭** | **✭** |  | **✭** | **✭** | **✭** | **7** |
| **Furuya**  **2009** | **✭** | **✭** | **✭** | **✭** | **✭✭** |  | **✭** | **✭** | **8** |
| **Hartikka**  **2005** | **✭** | **✭** | **✭** | **✭** |  | **✭** | **✭** | **✭** | **7** |
| **Ferrari**  **2005** | **✭** | **✭** | **✭** | **✭** | **✭** | **✭** | **✭** | **✭** | **8** |
| **Mizuguchi**  **2004** | **✭** | **✭** | **✭** | **✭** |  | **✭** | **✭** | **✭** | **7** |

**TableS2 Quality scores of included studies according to Agency for Healthcare Research and Quality (AHRQ) for cross-sectional studies.**

| **Author (Year)** | **①** | **②** | **③** | **④** | **⑤** | **⑥** | **⑦** | **⑧** | **⑨** | **⑩** | **(11)** | **Total Score** | **Quality level** |
| --- | --- | --- | --- | --- | --- | --- | --- | --- | --- | --- | --- | --- | --- |
| Inass M,2023 | 1 | 1 | 1 | 1 | 1 | 1 | 0 | 1 | 1 | 1 | 1 | 10 | High |
| Liu,2010 | 1 | 1 | 1 | 1 | 1 | 1 | 1 | 1 | 1 | 1 | 1 | 11 | High |

Description of AHRQ points：N stands for not sure；(1) Whether the source of the data is clear; (2) Whether the inclusion and exclusion criteria of the exposed group and the non-exposed group are listed; (3) Whether the time period for identifying osteoporosis patients is given; (4) Whether the study subjects are continuous; (5) Whether the subjective factors of the evaluator mask other aspects of the research subject; (6) Describe any assessments performed to ensure quality; (7) Explain the reasons for excluding any osteoporosis patients from the analysis; (8) Describe how to evaluate and/or control for confounding factors; (9) Whether it explains how to deal with missing data;(10) Summarizes the response rate of osteoporosis patients and the completeness of data collection; (11)Description of follow-up
